# The influence of heart disease on characteristics, quality of life, use of health resources, and costs of COPD in primary care settings

**DOI:** 10.1186/1471-2261-10-8

**Published:** 2010-02-18

**Authors:** Javier de Miguel-Díez, Pilar Carrasco-Garrido, Javier Rejas-Gutierrez, Antonio Martín-Centeno, Elena Gobartt-Vázquez, Valentín Hernandez-Barrera, Angel Gil de Miguel, Rodrigo Jimenez-Garcia

**Affiliations:** 1 Department of Pneumology. Hospital General Universitario Gregorio Marañón, Madrid, Spain; 2 School of Health Sciences, Universidad Rey Juan Carlos, Alcorcón (Madrid), Spain; 3 Department of Health Outcomes Research, Medical Unit, Pfizer España, Alcobendas (Madrid), Spain; 4 Medical Unit, Pfizer España, Alcobendas (Madrid), Spain; 5 Medical Department, Boehringer Ingelheim España, San Cugat del Vallés (Barcelona), Spain

## Abstract

**Background:**

To evaluate the influence of heart disease on clinical characteristics, quality of life, use of health resources, and costs of patients with COPD followed at primary care settings under common clinical practice conditions.

**Methods:**

Epidemiologic, observational, and descriptive study (EPIDEPOC study). Patients ≥ 40 years of age with stable COPD attending primary care settings were included. Demographic, clinical characteristics, quality of life (SF-12), seriousness of the disease, and treatment data were collected. Results were compared between patients with or without associated heart disease.

**Results:**

A total of 9,390 patients with COPD were examined of whom 1,770 (18.8%) had heart disease and 78% were males. When comparing both patient groups, significant differences were found in the socio-demographic characteristics, health profile, comorbidities, and severity of the airway obstruction, which was greater in patients with heart disease. Differences were also found in both components of quality of life, physical and mental, with lower scores among those patients with heart disease. Higher frequency of primary care and pneumologist visits, emergency-room visits and number of hospital admissions were observed among patients with heart diseases. The annual total cost per patient was significantly higher in patients with heart disease; 2,937 ± 2,957 vs. 1,749 ± 2,120, p < 0.05. Variables that were showed to be independently associated to COPD in subjects with hearth conditions were age, being inactive, ex-smokers, moderate physical exercise, body mass index, concomitant blood hypertension, diabetes, anxiety, the SF-12 physical and mental components and per patient per year total cost.

**Conclusion:**

Patients with COPD plus heart disease had greater disease severity and worse quality of life, used more healthcare resources and were associated with greater costs compared to COPD patients without known hearth disease.

## Background

Chronic obstructive pulmonary disease (COPD) is a process characterized by the presence of a chronic and poorly reversible obstruction of the airflow, related to an abnormal inflammatory reaction, primary against tobacco smoke [1]. In spite of the multiples campaigns against tobacco consumption conducted in recent years, nowadays, COPD is still an important problem of public health, with constantly increasing prevalence and mortality rates. Thus, in Spain, for instance, a prevalence of 9.1% has been described in subjects between 40 and 70 years of age [2], and in Latin America the prevalence ranges between 7.8% in Mexico City and 19.7% in Montevideo [3]. Concerning mortality, COPD represents the fourth cause of death, both, in Spain and worldwide [1].

Different studies have suggested that subjects with this condition are at an increased risk of cardiovascular morbidity and mortality, which cannot exclusively be attributed to the fact of sharing the habit of smoking as an etiological factor [4-6]. In fact, it has been demonstrated that an impaired pulmonary function constitutes a risk factor for cardiac mortality independent from the smoking habit, as important as the total cholesterol level [7]. Furthermore, several studies have shown that a substantial percentage of deaths among patients with mild COPD are the result of cardiovascular complications [8]; and in a wide epidemiologic study, an increase of cardiovascular mortality was observed, mainly in subjects with COPD under 65 years of age [4]. In this sense, systemic inflammation has been postulated to play a concurrent role in the pathogenesis and natural history of both diseases [9]. Heart conditions have also been related with smoking that could lead to systemic inflammation, then causing both health diseases.

Despite these data, the importance of establishing a correct diagnosis and scoring the seriousness of both processes combination is frequently underestimated [10]. In fact, limited information is available on the real influence of the presence of heart disease in patients with COPD, mainly in primary care clinical practice, where the percentage of patients with mild or moderate COPD is very high. Relative to this fact, some authors have questioned whether the results of typical clinical trials conducted in patients with COPD can be extrapolated to "real life" patients [11].

The primary objective of this study was to evaluate the influence of heart disease on clinical characteristics, quality of life, use of healthcare resources, and calculation of related costs of patients with COPD attended at primary care settings under common clinical practice conditions.

## Methods

### Design and Study Population

This study was part of the EPIDEPOC study, a descriptive, epidemiological, observational, and multicentric project, conducted at primary care settings with the objective of estimating the use of health resources, and assesses the quality of life of patients with stable COPD [12].

Patient recruitment and calculation of the sample size corresponded to that conducted in the EPIDEPOC study. For calculation of sample size, a cluster design was used, considering 3 types of variables: health centres, physicians, and medical records. As the health centres were considered to be homogeneous and representatives of the Spanish geographical population, the medical record was chosen as the unit of study and the prescriber as the cluster. A previous study in a large cohort of 1,510 primary care patients found that the average annual cost per patient varied widely, with an estimated standard deviation of €3,407 [13]. Assuming a precision of €90, 5,505 medical records needed to be evaluated. If the effect of the cluster design is also taken into account, i.e., the loss of efficacy from the use of clusters, assuming a correlation of 0.3 and a cluster size of 5, a total of 2,422 prescribers and 12,111 medical records would be required. The subjects were consecutively included by primary care physicians in all the Spanish Autonomous Communities, with a distribution proportional to the population in each Community. The patients were recruited during a period of three months (from January 1 to March 31, 2003).

Patients of both genders, over 40 years of age, and with a diagnosis of COPD made at least 12 months before study initiation were included. The diagnosis of COPD was made according to the Spanish Society of Pneumology and Thoracic Surgery (SEPAR) criteria, based on the evidence, from a forced spirometry, of a forced expiratory volume during the first second (FEV_1_) lower than 80% of the reference value, and a ratio FEV_1_/forced vital capacity (FVC) lower than 0.7 after a bronchodilator test. Depending on the FEV_1_value, the seriousness of the disease was qualified into three levels: mild (FEV_1_: 60-80% of the reference value), moderate (FEV_1_: 40-59% of the reference value), and serious (FEV_1_: under 40% of the reference value), in accordance with the SEPAR Criteria [14].

Subjects with neurological or psychiatric diseases that could hamper the assessments during the study were excluded. Patients who had suffered an exacerbation of COPD within the previous month were excluded as well. The exacerbation was defined as the impairment of the patient's clinical status characterized by increased expectoration, the presence of purulence in the sputum, increase of baseline dyspnea, or any combination of these symptoms [15].

The Ethics Committee of the *Fundación Hospital Alcorcón *approved the study, and all patients gave their oral consent to participate.

### Patients' Evaluation

Patients attended only one visit, and socio-demographic, seriousness of the disease, and use of healthcare resources within the 12 previous months data from all patients were collected. Furthermore, associated comorbidities, including the presence or absence of heart disease, according to the ICD-9 diagnostic criteria, were recorded. The ICD-9 codes that were used to define the presence of heart disease were: 410-114 (ischemic heart disease) and 420-429 (other forms of heart diseases). Patients were divided in two groups (with heart disease and with no heart disease) according to this criterion. All patients were administered the SF-12 quality of life questionnaire, an abbreviated version of the SF-36 health questionnaire that contains 12 items [16,17]. These 12 items explain more than 90% of the variance of the physical and mental component scores of the SF-36. From them two scores can be calculated, the physical (PCS-12) and the mental (PCS-12) component summary, using a value of 50 with a standard deviation of 10 as reference population. In this study, the general Spanish adult population has been used as reference [18]. The SF-12 is scored from 0 (worst possible health quality of life) to 100 (best possible quality of life).

The use of health resources by patients was obtained from data recorded in medical records and through patients' interviews, in which the consumption of health resources within the 12 previous months was recorded. The collected health resources included the following: drugs, medical visits (including visits to emergency departments), complementary tests, hospitalizations, oxygentherapy, and other non-pharmacologic treatments and vaccines received. To obtain indirect costs, the "human capital" method was used. This method uses a basic hypothesis on the equivalence between the value of lost productivity and the associated salary for obtaining of this production. This is the loss of one working day implies a production loss equal to the salary the patient would have received that day. Information on employment and salaries was obtained from the Spanish National Statistics Institute [19]. For the study, the number of lost working days due to the disease within the previous year was recorded.

### Statistical aspects

SPSS 14.0 statistical pack for Windows was used for the analysis of data. Categorical variables were described through frequencies and percentages, and continue variables through means, standard deviations, and minimum and maximum values. A Pearson χ^2 ^test was used to analyze the relationship between qualitative variables. A Student t test for independent measures was used to calculate means differences between both groups. The results were adjusted for age and gender through the direct method, using the Spanish population in accordance with the year 2003 census as the reference population. Multivariate analysis using logistic regression was conducted to explore the impact of chronic heart disease on COPD. P values < 0.05 were considered statistically significant.

Annual costs were estimated by multiplying the unit cost of each resource (cost per treatment day for drugs, depending on the dose received) by the number of resources used within the 12 months previous to the study visit, and were expressed in annual costs per patient. Unit costs of drugs were obtained from the Pharmaceutical Catalogue of the General Council of Pharmacists' Colleges of Spain, while costs of other health resources were obtained from the information on costs of different procedures supplied by the managers of the Área 8 de Madrid, Hospital de Alcorcón, and Hospital de Móstoles.

## Results

A total of 10,711 patients with COPD were assessed. Information regarding patient background on hearth condition was collected in 9,390 subjects (87.6%). Among these patients, 1,770 (18.8%) had a concomitant heart disease; with 78% of them being males. Table 1 shows the socio-demographic characteristics of patients according to the presence of heart disease. Regarding tobacco consumption, the percentage of ex-smokers was higher among patients with heart disease (66.5%), compared to those patients without this comorbidity (56.1%), while the percentage of active smokers was significantly lower (11.6% versus 20.7%, p < 0.05). In general, differences were found between both patient groups in the practice of physical exercise, level of obesity, severity of the airflow obstruction, and comorbidities; in all cases differences were against patients with a past history of heart disease (Table 2). On the other hand, quality of life questionnaires scores were significantly lower among patients with associated heart disease in both SF-12 summary components, but particularly in the physical component; 30.7 ± 8.7 vs. 37.0 ± 9.8, p < 0.05 (Table 2).

**Table 1:** Socio-demographic characteristics of patients with COPD examined in function of the presence or the absence of associated heart disease

Parameter	Patients without heart disease (n = 7,620)	Patients with heart disease (n = 1,770)	p
Age (mean ± SD)	65.92 ± 9.56	73.73 ± 8.29	< 0.05
Gender: Male (%)	75.0	78.9	< 0.05
			
Labour status:			< 0.05
Inactive (%)	73.5	93.4	
Active (%)	26.5	6.6	
			
Level of education:			
No education (%)	19.4	26.7	< 0.05
Primary (%)	56.9	55.7	
Secondary (%)	18.2	13.4	
University (%)	5.6	4.1	

**Table 2:** Health profile, severity of the airflow obstruction according to the VEF_1_, comorbidities and quality of life according to the SF12 of patients with COPD examined in function of the presence or the absence of associated heart disease*.

Parameter	Patients without heart disease (n = 7,620)	Patients with heart disease (n = 1,770)	p
Tobacco consumption:			
Never (%)	23.3	21.9	< 0.05
Ex-smoker (%)	56.1	66.5	
Active smoker (%)	20.7	11.6	
Physical exercise:			
None (%)	30.4	40.7	< 0.05
Light (%)	64.7	57.3	
Moderate (%)	5.0	2.0	
Level of obesity**:			
Normal weight (%)	24.6	21.2	< 0.05
Overweight (%)	55.3	48.7	
Obesity (%)	20.1	30.2	
Severity of obstruction (VEF_1_):			
Mild	37.7	24.4	< 0.05
Moderate	53.3	53.3	
Serious	8.9	22.3	
Comorbidities:			
Blood hypertension (%)	40.8	64.3	< 0.05
Hypercholesterolemia (%)	37.7	44.5	< 0.05
Diabetes (%)	12.2	29.5	< 0.05
Gastroduodenal ulcer (%)	13.7	19.2	< 0.05
Depression (%)	10.9	16.3	< 0.05
Anxiety (%)	19.8	25.9	< 0.05
Quality of life (SF-12):			
Mental component (mean ± SD)	48.7 ± 10.8	46.5 ± 11.9	< 0.05
Physical component (mean ± SD)	37.0 ± 9.8	30.7 ± 8.7	< 0.05

* All percentages and means have been adjusted to age and gender using the direct method.

** Normal weight (BMI < 27), overweight (BMI ≥ 27 and < 30), and obesity (BMI ≥ 30). NS: not significant. SF-12 range: 0 (worst possible quality of life) - 100 (best quality of life).

Among subjects with heart disease, a greater consumption of anticholinergic drugs (88.6% vs. 83.9%, p < 0.05), as well as teophyllines (15.6% vs. 11.3%, p < 0.05), and steroids, both inhaled (28.5% vs. 21.5%, p < 0.05) and oral (7.4% vs. 5.3%, p < 0.05) was observed. Conversely, no significant differences in the use of β_2_-adrenergic agonists or mucolytic drugs were found. Concerning the past history of vaccinations, patients with heart disease had been more frequently submitted to anti-influenza vaccination within the last campaign or anti-pneumococcal vaccination at any time (Table 3).

**Table 3:** Present drug treatment and vaccines of patients with COPD examined in function of the presence or the absence of associated heart disease

Treatment	Patients without heart disease (n = 7,620)	Patients with heart disease (n = 1,770)	p
Short-action β2-adrenergic agonists (%)	39.3	42.3	NS
Long-action β2-adrenergic agonists (%)	9.2	10.4	NS
Anticholinergic drugs (%)	83.9	88.6	< 0.05
Teophylline (%)	11.3	15.6	< 0.05
Inhaled steroids (%)	21.5	28.5	< 0.05
Oral steroids (%)	5.3	7.4	< 0.05
Mucolytic drugs (%)	8.5	10.2	NS
Antiinfuenza vaccine during the previous campaign (%)	86.7	91.0	< 0.05
Antipneumococcal vaccine at any time (%)	31.6	39.2	< 0.05

* All percentages and means have been adjusted to age and gender using the direct method.

NS: not significant.

Patients with heart disease had consumed significantly more resources than those with no COPD-associated heart disease. Therefore, mean numbers of primary care and pneumology consultations, visits to emergency departments, hospitalizations, and duration of hospital admissions were significantly greater in the first patient subgroup (Table 4). Due to the resulting cost, the prolonged length of hospital stay of patients with heart disease should be highlighted; 11.82 ± 14.99 vs. 6.09 ± 10.32 (p < 0.05, Table 4).

**Table 4:** Use of health resources during the previous year in patients with COPD in function of the presence or the absence of associated heart disease*.

Health resources	Patients without heart disease (n = 7,620)	Patients with heart disease (n = 1,770)	p
No. of visits to the primary care doctor	6.48 ± 5.51	7.75 ± 6.59	< 0.05
No. of visits to the pneumology clinics	1.36 ± 1.40	1.79 ± 2.01	< 0.05
No. of visits to emergency departments	1.83 ± 0.83	2.10 ± 0.94	< 0.05
No. of hospital admissions	0.40 ± 1.10	0.90 ± 1.50	< 0.05
Length of hospital stay (days)	6.09 ± 10.32	11.82 ± 14.99	< 0.05

* Values are expressed as mean ± Standard deviation. All means have been adjusted to age and gender using the direct method. NS: not significant.

Table 5 shows the costs of COPD by presence of an associated heart disease. The annual total cost per patient was significantly higher among patients with heart disease than those without the condition; 2,937.19 ± 2,956.69 vs. 1,749.25 ± 2,120.28, p < 0.05. This increased total cost was due to significantly higher costs in all cost components, included indirect costs; however, due patients' age, and to the fact of most patients were retired, indirect costs had a limited impact. Due to the increased length of hospital stay, the most expensive component, differences in this component had the greatest magnitude as well; 1,494.44 ± 2,321.38 vs. 658.13 ± 1,610.29 (p < 0.05, Table 5).

**Table 5:** Annual costs per patient of health resources used in patients with COPD in function of the presence or the absence of associated heart disease.

Cost component (€)	Patients without heart disease (n = 7,620)	Patients with heart disease (n = 1,770)	p
Visits to PC doctor	104.63 ± 93.01	124.37 ± 111.22	< 0.05
Visits to specialist doctor	88.87 ± 98.51	119.42 ± 140.80	< 0.05
Visits to the emergency department	127.00 ± 188.77	184.18 ± 229.74	< 0.05
Hospitalization	658.13 ± 1610.29	1494.44 ± 2321.38	< 0.05
Diagnostic tests	122.62 ± 135.92	164.78 ± 194.29	< 0.05
Drugs	483.57 ± 406.76	575.71 ± 441.79	< 0.05
Oxygentherapy	70.66 ± 258.76	215.53 ± 420.49	< 0.05
Vaccination			
influenza	0.22 ± 0.60	0.14 ± 0.49	< 0.05
Pneumococcal	4.50 ± 6.77	5.62 ± 7.14	< 0.05

Sick leave days	76.61 ± 297.42	38.33 ± 281.31	< 0.05

**Total cost**	**1,749.25 ± 2,120.28**	**2,937.19 ± 2,956.69**	**< 0.05**

* All means have been adjusted to age and gender using the direct method. PC = Primary care.

Variables that, as per the results of multivariate logistic regression, were showed to be independently associated to COPD in subjects with hearth conditions were age, being inactive, ex-smokers, moderate physical exercise, body mass index, concomitant blood hypertension, diabetes, anxiety, the SF-12 physical and mental components and per patient per year total cost (table 6).

**Table 6:** Variables independently associated to COPD among chronic heart disease patients using multivariate logistic regression analysis.

	OR	IC 95%
	
Age	1.06	(1.05-1.07)
Labour status:		
Active	1	-
Inactive	1.57	(1.43-1.75)

Tobacco consumption:		
Never	1	-
Ex-smoker	1.47	(1.22-1.76)
Active smoker	1.16	(0.89-1.50)
Physical exercise:		
None	1	-
Light	1.10	(0.94-1.29)
Moderate	1.72	(1.03-2.87)
Level of obesity:		
Normal weight	1	-
Overweight	1.04	(0.86-1.26)
Obesity	1.46	(1.18-1.81)
Comorbidities:		
Blood hypertension	1.85	(1.59-2.15)
Diabetes	169	(1.42-2.01)
Anxiety	1.23	(1.03-1.48)
Quality of life (SF-12):		
Mental component	0.99	(0.98-0.99)
Physical component	0.96	(0.95-0.96)
		
Annual total cost per patient	1.01	(1.01-1.02)

## Discussion

The presence of comorbidities is frequent among patients with COPD, although different studies have shown a wide range of prevalence rates [1,20-23], possibly due to differences in patient selection criteria and severity of the disease among different studies. In our study, we found a prevalence of hearth disease of 18.8%. Unfortunately, the presence of both conditions is dreadful, and, as demonstrated in this study, significantly contributes to the worse quality of life, and increased resources consumption and costs of patients with COPD.

The causal relationship between COPD and heart disease has been traditionally attributed to cigarette consumption, but the exact mechanism has been broadly studied recently [24]. Several epidemiologic studies have indicated the importance of systemic inflammation in the pathogenesis of arteriosclerosis and cardiovascular events, and recent studies have suggested that COPD patients have a prominent systemic inflammatory response [25-30]. For instance, in patients with COPD an increased C reactive protein (PCR) level, a known marker of systemic inflammation, has been detected [31-34]. Since an increased CRP is a risk factor for cardiovascular diseases [35], the pathogenesis of both processes may possibly start with an increased systemic inflammation.

After recognizing the connection between pulmonary and heart diseases, both conditions related from a pathophysiologic point of view, it is important to determine the characteristics of patients with COPD and an associated heart disease as a comorbidity. In this study we observed that these patients, compared to those with COPD but no associated heart disease, require more respiratory drugs, have a worse quality of life, consume more resources, and generate greater expenses.

Patients with COPD consume medicines stimulating their cardiovascular system, as are sympathomimetic drugs. These pharmacological agents may result in increased blood pressure and cardiac frequency, abnormalities that might lead to the development of a cardiovascular event. The stimulation of the cardiovascular system, might also lead to the occurrence of an arrhythmia, some of them potentially life-threatenning [4]. In this sense, in a recent metanalysis Salpenter et al [36] evidenced an increased incidence of tachycardia and hypokalemia among patients with COPD who took β_2_-adrenergic agonists. This suggests that these abnormalities might contribute to the increased number of cardiovascular deaths among these patients. However, these results have not been confirmed in later studies. Therefore, in the TORCH study (TOwards a Revolution in COPD Health), in which over 6,000 patients with COPD were randomized to receive salmeterol, a salmeterol-fluticasone combination, or placebo, no increases of overall mortality or cardiovascular adverse events were shown in the salmeterol group compared to the other groups [37]. In our study, no differences have been found in the use of β_2_-adrenergic agonists among patients with COPD, regardless of the presence or the absence of heart disease; however, an increased use of anticholinergic drugs, bronchodilator drugs with lover cardiovascular effects, was observed among patients with heart disease. Another drug class that might contribute to the occurrence of tachyarrhythmia, even in the absence of treatments increasing potassium levels, is the methylxantines drug class [38]. However, in our study, patients with heart disease were taking this type of bronchodilators more frequently, possibly due to a greater severity of COPD compared to patients with no associated heart disease. The same was observed for the use of steroids, in spite of previous studies demonstrating a relationship between the use of oral steroids and the development of auricular fibrillation and ventricular arrhythmia [38,39].

Relative to associated diseases, the presence of comorbidities constitutes an important factor determining the quality of life of patients with COPD, regardless the LVEF [40,41], and the use of health resources [42]. However, the specific influence of heart disease on the quality of life of patients with COPD attending primary care settings had not been assessed in previous studies. Our study indicated worse scores in both components, physical and mental, of quality of life questionnaire among patients with COPD and associated heart disease.

On the other hand, comorbidities are factors predicting an increased use of health resources [43]. In particular, the presence of a cardiovascular disease has demonstrated to be an important cause of hospitalization among patients with COPD [6]. In addition, this study demonstrated greater lengths of hospital stay among patients with COPD and associated heart disease, compared to those patients with no cardiac comorbidity. Increased numbers of primary care, pneumologist, and emergency department visits was evidenced as well for the first patient group. These results contrast with those obtained in another recent study conducted in the UK, demonstrating a negative correlation between the presence of comorbidity and the number of visits to primary care settings [44]. Nevertheless, the correlation with the use of the second level of care was positive; this suggesting that patients with a more serious disease might have a closer link to this domain.

Comorbidities are factors predicting increased costs of COPD, even more than parameters of pulmonary function. However, limited studies are available assessing the financial impact of comorbidities management in COPD patients. Our study, evidenced a significant increase of direct costs among patients with COPD and associated heart disease, compared to patients with no associated heart disease. Furthermore, the presence of heart disease has demonstrated to be an independent factor determining the cost of COPD [45]. In contrast, a recent study conducted in Spain, which obtained a similar prevalence of cardiovascular diseases, could not demonstrate an increase of overall costs of COPD as a consequence of this comorbidity [46].

Nevertheless, the authors underline some limitations that force to be cautious when generalizing the results, as the study design, possible inconsistencies among participant professionals, and coordination between assistance levels, obstructing the measurement of cost-efficient actions. The two-stage sampling may be a limitation, since the first selection of primary care physicians was followed by a systematic patient's selection through a procedure that might entail biases. The participation of physicians was voluntary, which could mean that these professionals were particularly interested in this disease, and that this would have an effect on the follow-up of the study patients. The high rate of participating physicians might have reduced the magnitude of this bias, should it exist. Biases might also be present in subjects' selection. It should be taken into account that not all COPD patients are treated in primary care centres, or even in the public health care setting. In this sense, those patients with more comorbidities (including heart disease) are more likely of being treated in the specialized care setting. Similarities between socio-demographic characteristics of our patients and those of other Spanish-based COPD studies are in favour of the representative nature of the sample [2,47,48]. Finally, we computed indirect costs in working population as well. In this study, the inability to perform daily activities as a consequence of the disease in retired subjects was not considered a cost.

Further limitations to be taken into a count refer to recall bias of data collection. Retrospective data collection of resource use is known to have this type of bias problems. Moreover, primary care clinical records are also known to be a substandard source, as a lot of the resources are not written down in the patient chart. On the other hand, higher prevalence of comorbidities may also mean that COPD patients with heart disease are more closely monitored (workup bias) so has more diagnosis.

## Conclusion

In conclusion, this study has shown that patient with COPD and a heart disease as a comorbidity shows worse quality of life, higher healthcare resources use, increased consumption of drugs, and more disease management costs. Nevertheless, and due to the fact that this was a cross sectional study, a cause-effect association can not be established but a possible association only which was the poorer outcomes with concurrent heart disease. However, while patients with heart disease were more likely to have other health factors playing a role, poorer outcomes could certainly not to be the cause. Additional studies providing new data on the pathogenesis and management of patients with both conditions are needed, with the purpose of trying to improve quality of life as well as survival of patients with COPD.

## Competing interests

JRG and AMC are employees at Pfizer Spain and EGV is employee at Boehringer Ingelheim SA. The other authors have not any conflict of interest. This study has been funded by an unrestricted grant from Pfizer Spain and Boehringer Ingelheim SA.

## Authors' contributions

PCG, JRG, VHB, AGM and RJG have made substantive intellectual contributions to conception and design, acquisition of data and analysis and interpretation of data. PCG, JRG, JMD, AMC, AGM, and EGV have been involved in drafting the manuscript and revising it critically for important intellectual content. All authors have given final approval of the version to be published.

## Pre-publication history

The pre-publication history for this paper can be accessed here:

http://www.biomedcentral.com/1471-2261/10/8/prepub
